# Annealing-Driven Modifications in ZnO Nanorod Thin Films and Their Impact on NO_2_ Sensing Performance

**DOI:** 10.3390/mi16070778

**Published:** 2025-06-30

**Authors:** Sandip M. Nikam, Tanaji S. Patil, Nilam A. Nimbalkar, Raviraj S. Kamble, Vandana R. Patil, Uttam E. Mote, Sadaf Jamal Gilani, Sagar M. Mane, Jaewoong Lee, Ravindra D. Mane

**Affiliations:** 1 Department of Physics, Jaysingpur College, Jaysingpur, Shivaji University, Kolhapur 416101, Maharashtra, India; 2 Department of Physics, Bhogawati Mahavidyalaya, Kurukali, Shivaji University, Kolhapur 416001, Maharashtra, India; 3 Sharad Institute of Technology, Polytechnic, Yadrav, Ichalkaranji 416121, Maharashtra, India; 4 Department of Chemistry, Bhogawati Mahavidyalaya, Kurukali, Shivaji University, Kolhapur 416001, Maharashtra, India; 5 Department of Physics, Shrimant Babasaheb Deshmukh Mahavidyalaya, Atpadi, Shivaji University, Kolhapur 415301, Maharashtra, India; 6 Department of Pharmaceutical Sciences, College of Pharmacy, Princess Nourah bint Abdulrahman University, P.O. Box 84428, Riyadh 11671, Saudi Arabia; sjglani@pnu.edu.sa; 7 Department of Fiber System Engineering, Yeungnam University, Gyeongsan 38541, Gyeongbuk, Republic of Korea

**Keywords:** ZnO nanorod array, annealing temperature variation, growth orientation, NO_2_ sensor

## Abstract

This research examines the effect of annealing temperature on the growth orientation of zinc oxide (ZnO) nanorods and its subsequent influence on NO_2_ gas sensing efficiency. Zinc oxide (ZnO) nanorods were synthesized using the chemical bath deposition method, followed by annealing at 300, 400, and 500 °C. Diffraction analysis confirmed that both non-annealed and annealed ZnO nanorods crystallize in a hexagonal wurtzite structure. However, increasing the annealing temperature shifts the growth orientation from the c-axis (002) toward the (100) and (101) directions. Microscopy images (FE-SEM) revealed a reduction in nanorod diameter as the annealing temperature increases. Optical characterization using UV–visible and photoluminescence spectroscopy indicated shifts in the band gap energy and emission properties. Contact angle measurements demonstrated the hydrophobic nature of the films. Gas sensing tests at 200 °C revealed that the ZnO thin film annealed at 400 °C achieved the highest NO_2_ response of 5.88%. The study highlights the critical role of annealing in modifying the crystallinity, growth orientation, and defect states of ZnO thin films, ultimately enhancing their NO_2_ detection capability.

## 1. Introduction

Metal oxide-deposited films are widely utilized in gas sensing applications due to their exceptional sensitivity, selectivity, and long-term stability. These materials show varying responsiveness to specific gases, making them crucial for applications that ensure human and environmental safety, as well as for medical diagnostics. Their ability to operate under different conditions and detect various gases ensures their extensive use in modern sensing technologies. Different metal oxides demonstrate unique sensing properties, making them suitable for detecting specific gases. For example, tin dioxide (SnO_2_) is one of the most commonly used metal oxides in commercial gas sensors due to its high sensitivity to gases such as methane (CH_4_), carbon monoxide (CO), and hydrogen (H_2_) [1,2]. SnO_2_ operates efficiently at elevated temperatures and is capable of detecting both reducing and oxidizing gases, making it a versatile choice in gas sensor technology. Titanium dioxide (TiO_2_), another widely studied metal oxide, possesses excellent chemical stability, allowing it to function effectively in harsh environmental conditions. It is particularly sensitive to nitrogen oxides (NO_x_), ammonia (NH_3_), and hydrogen (H_2_). However, due to its wide band gap, TiO_2_-based sensors often require ultraviolet (UV) light activation to enhance their sensing performance [3]. This requirement makes TiO_2_ sensors more suitable for controlled environments where UV activation can be consistently provided. Copper oxide (CuO), a p-type semiconducting material, is known for its ability to detect reducing gases. Carbon monoxide (CO), ammonia (NH_3_), and hydrogen sulfide (H_2_S) are the key examples of reducing gases. Among these gases, CuO demonstrates exceptional sensing performance for H_2_S, offering high sensitivity and selectivity specifically for this gas. As a result, CuO is a highly valuable material for gas sensing applications requiring precise detection of toxic gases [4]. Cobalt oxide (Co_3_O_4_), another p-type semiconductor, is commonly used in gas detection. Gas sensors based on Co_3_O_4_ are particularly efficient at detecting methane (CH_4_), carbon dioxide (CO_2_), and carbon monoxide (CO). One of its key advantages is its ability to operate at lower temperatures while maintaining high sensitivity, making it suitable for various environmental monitoring applications [5].

Aligning with these metal oxides, ZnO is a highly versatile semiconducting material extensively employed in electronic devices due to its exceptional properties [6]. Significant optical gain (300 cm^−1^), high excitonic binding energy, strong cohesive energy (1.89 eV), a broadband gap of 3.37 eV, and remarkable mechanical and thermal stability are the key features associated with this material. These characteristics make it an ideal candidate for detecting gases such as hydrogen (H_2_), carbon monoxide (CO), and ethanol, along with applications in optoelectronics, sensors, and photovoltaics. Furthermore, the performance of ZnO can be significantly enhanced through morphology modification and doping with other materials. By altering its structural characteristics, such as particle size, shape, and surface area, the material’s charge transport, optical absorption, and surface reactivity can be optimized for specific applications. Doping with elements like Al, Ga, In, or transition metals further refines its electrical conductivity, carrier concentration, and defect states, making it suitable for advanced applications in optoelectronics, gas sensing, catalysis, and energy storage [7,8,9]. The modification of physicochemical properties and the attainment of desired characteristics in ZnO are highly dependent on the synthesis conditions, including the deposition method, temperature, duration, precursor concentration, and ambient environment. Several reports in the literature discuss different synthesis techniques for ZnO thin-film deposition [10,11,12,13,14,15], each offering distinct advantages based on the desired film properties, deposition conditions, and intended applications.

Among various synthesis methods, the aqueous solution-based synthesis of ZnO films is a simple and cost-effective alternative to complex vapor deposition techniques. Chemical bath deposition (CBD) is widely used for thin-film fabrication due to its advantages, including large-area deposition, low-temperature processing, and the elimination of high-vacuum requirements, making it an efficient and economical choice [16,17]. Furthermore, oxygen vacancies (O_v_) are key to determining the carrier concentration in n-type ZnO nanomaterials. The annealing process, influenced by temperature, affects these vacancies and the material’s electrical properties. Intrinsic defects, linked to the deposition technique, can be optimized through post-deposition annealing, which improves crystallinity and reduces defects, enhancing the quality of ZnO thin films. Optimizing the annealing temperature is essential for achieving high crystallinity and minimal defects while maintaining morphology [18].

Several research groups have explored ZnO nanomaterials for gas sensing applications. I. A. Pronin et al. compared conventional and photo-annealing methods for ZnO thin films, testing their response to alcohol vapors [19]. M. Hjiri et al. studied the NH_3_ gas sensing properties of Ca-doped ZnO nanoparticles synthesized via sol–gel techniques [20]. S. Ozturk et al. investigated Pd and Pt-coated ZnO nanorods for H_2_ detection, finding superior sensor responses with Pd coatings [21]. Sanjay Kumar et al. synthesized ZnO microstructures with Pt nanoparticles for enhanced H_2_ gas sensing [22]. L. Arunraja et al. optimized ZnO nanocomposites through annealing, improving responses, recovery times, and stability [23]. D. T. Speaks studied the effect of annealing on ZnO and ZnO: Al thin films, identifying 350 °C as the minimum temperature for high-quality films [24]. Beyond gas sensing, the influence of annealing temperature on photocatalytic performance was investigated by A. Umar et al. [25]. They synthesized ZnO nanoparticles and examined how annealing temperature affected their efficiency in degrading DR-23 dye. Similarly, A. Zaier et al. [26] employed thermal evaporation to fabricate ZnO thin films, annealing them between 200 and 500 °C. Their study analyzed structural, optical, and electrical changes, revealing that both the band gap and resistivity increased with rising annealing temperature. These studies emphasize the significant impact of annealing temperature on ZnO fabrication, influencing its essential properties and suitability for various applications.

In line with with the above discussion, this study focuses on annealing-driven modifications in ZnO nanorod thin films and investigates their impact on NO_2_ sensing performance. ZnO nanorods synthesized using the CBD method underwent annealing at different temperatures while keeping the duration constant. The alterations in growth orientation and their influence on gas sensing performance were systematically examined, along with a detailed assessment of optical and luminescence properties. Gaining insight into these relationships will aid in developing more efficient and reliable ZnO-based sensors for NO_2_ detection, enhancing safety-related applications aimed at protecting both the environment and human health.

## 2. Materials and Methods

### 2.1. Chemicals

AR-grade chemicals with purity of 99.9%, including Zn(CH_3_COO)_2_·2H_2_O, NH_4_OH, and C_6_H_12_N_4,_ were procured from Thomas Bakers India Limited and used as received.

### 2.2. Deposition of ZnO Thin Films

**Figure 1:**
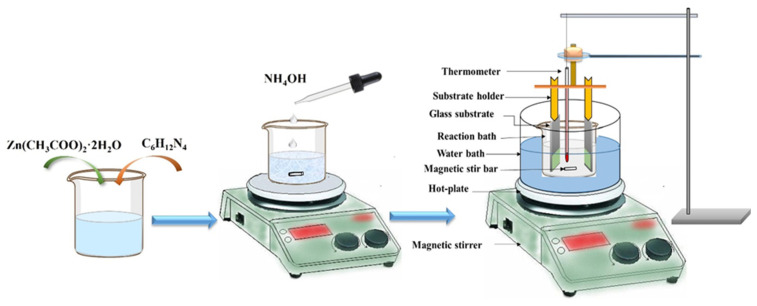
Thin-film deposition setup.

To grow vertical nanorods of ZnO on a glass substrate, a chemical bath deposition process was undertaken. The deposition setup is illustrated in Figure 1. Prior to commencing the growth procedure, glass slides underwent thorough cleansing by being fully submerged in boiling 0.2 M chromic acid. Afterward, they were rinsed with double-distilled water (DDW) and subsequently treated with ultrasound for 15 min. Finally, the glass slides were washed using acetone and then positioned to grow the ZnO. Uniform and adherent ZnO films were synthesized by immersing the substrate in a 100 mL beaker filled with an equimolar aqueous solution of zinc acetate dihydrate and hexamethylenetetramine (HMTA) in DDW. The deposition process was carried out at 80 °C for an interval of 2 h. During the deposition process, the solution pH was maintained at 12, and to achieve this condition, aqueous ammonia was poured dropwise. The deposited thin-film samples were drawn from the reaction solution, washed with deionized (DI) water, and dried under airflow. Finally, these thin films were air-annealed for 10 min, with temperature variations of 300, 400, and 500 °C. These annealed films and one annealed film functioning as a gas sensor were used.

#### 2.2.1. Characterizations

Crystallographic analysis was conducted using X-ray diffraction (AXS D8 Advance, Bruker, Karlsruhe, Germany) with Cu-Kα anode of wavelength 1.54 Å. Surface morphology was studied through a field emission scanning electron microscope (FE-SEM) (Tescan, Drno, Czech Republic, MIRA3 LMH). Surface chemical analysis and elemental states were confirmed using X-ray photoelectron spectroscopy (Thermo Fisher Scientific (K-Alpha), Seoul, Republic of Korea). The molecular bonding and structural attributes of ZnO nanomaterials were examined via an FT-IR spectrometer (FT-IR4600, Jasco, Tokyo, Japan). To assess the optical characteristics, a UV-Vis spectrophotometer (V770, Jasco, Tokyo, Japan) was employed. Luminescence behavior was explored using a photoluminescence (PL) spectrophotometer (FP8200, Jasco, Tokyo, Japan). Furthermore, contact angle measurements of ZnO films were performed using the sessile drop method, where a water droplet was analyzed with a microscope-integrated goniometer.

#### 2.2.2. Gas Sensing Performance Measurement

The gas sensing performance of all ZnO films, i.e., films fabricated without annealing and with annealing at different temperatures, was investigated using dual-probe gas detection apparatus. A silver paste was applied to the thin film to act as an electrode in order to assess the gas detection potential. For the gas sensing studies, the apparatus was placed inside a sealed test unit, referred to as the sensor device, through which the target gas was introduced within a specific temperature range. A dual-probe gas sensing setup has a Keithley source meter that detects changes in the resistivity film under investigation in the atmospheric air and upon the insertion of a target gas. The gas response (*R_s_*) was determined by analyzing the resistance of the thin film in the absence (*R_a_*) and presence (*R_G_*) of the gas while varying gas concentrations within the sensor device.

(1) $$R_{s}=\frac{R_{G}}{R_{a}}$$

## 3. Results and Discussion

**Figure 2:**
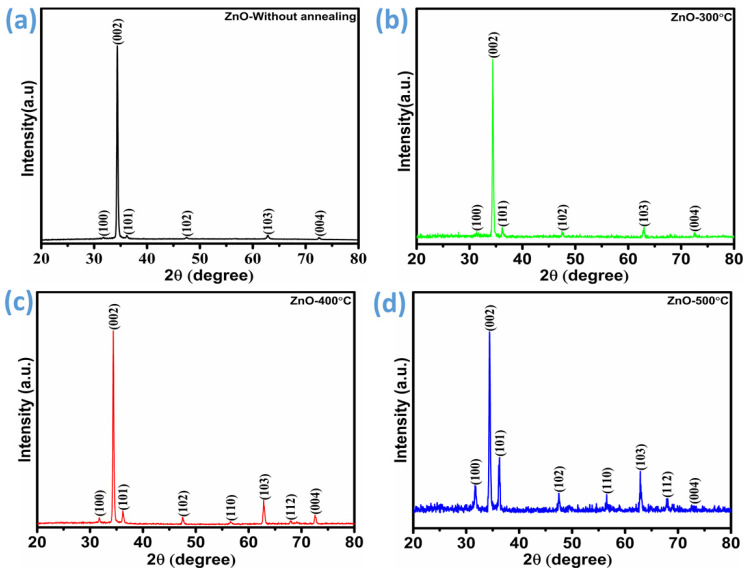
XRD patterns of ZnO thin films: (**a**) as-deposited and (**b**–**d**) annealed samples at 300 °C, 400 °C, and 500 °C.

To thoroughly assess crystallinity, the diffraction patterns of ZnO films were examined across the 2*θ* range of 20° to 80°. These analyses included films both before and after annealing at different temperatures. Figure 2a presents the diffraction pattern of the unannealed ZnO film, while Figure 2b–d display the results for films subjected to annealing at 300 °C, 400 °C, and 500 °C, respectively. These patterns confirm that the ZnO nanomaterial exhibits a polycrystalline nature, aligning well with the reference data ICDD card no. 36-1451. Characteristic diffraction peaks are observed at 2*θ* values of 31.79°, 34.38°, 36.17°, 47.50°, 56.62°, 62.88°, and 72.51°, which correspond to the (100), (002), (101), (102), (110), (103), (112), and (004) crystal planes, respectively. A detailed analysis of the diffraction patterns revealed that the (110) and (112) crystal planes, positioned at 2*θ* = 56.62° and 2*θ* = 68°, became evident when the annealing temperature reached 400 °C and 500 °C. In contrast, this peak remained undetectable in samples processed at lower temperatures and in unannealed ZnO. The (002) crystal plane was observed to be the most prominent feature in all diffraction patterns, indicating preferential nanorod growth along the c-axis. The significant kinetic energy associated with this orientation likely facilitates its rapid formation compared to other planes [27]. However, with the introduction of annealing, the (100) and (101) planes also exhibited noticeable enhancement. This suggests a shift in the preferred growth orientation, likely due to enhanced atomic diffusion and recrystallization effects that took place due to annealing [25,28,29]. Additionally, variations in film thickness, crystallization quality, and density significantly influence the shift in orientation [30]. This intuitive change in orientation can significantly impact the material’s physical properties, including its electronic band structure, optical absorption characteristics, and carrier mobility. The shift in the preferred crystal orientation from the (002) plane to the (100) and (101) planes exerts a significant impact on both the surface characteristics and the gas sensing behavior of the material. In many layered materials, such as ZnO, the (002) plane is typically associated with closely packed atomic layers and low surface energy, which can result in lower surface reactivity. Conversely, the (100) and (101) planes often exhibit higher surface energy and a greater density of unsaturated atoms or dangling bonds, which enhance their chemical reactivity [31,32]. Overall, it can be noted that annealing at a certain temperature induces structural rearrangements, leading to the initiation of the crystallite reorientation process. These alterations can significantly impact the gas sensing performance of ZnO by modifying surface chemistry, adsorption sites, and charge carrier dynamics [33,34]. Therefore, the annealing temperature plays a critical role in optimizing the structural and functional properties of ZnO, ensuring its effectiveness in gas sensing applications.

The key parameters obtained from the XRD analysis are summarized in Table 1, highlighting the influence of annealing temperature. A slight variation in crystallite size is observed with increasing annealing temperature. The interplanar distance *d_hkl_* of ZnO thin films was evaluated by Bragg’s law.

(2) $$2\;d_{hkl}\;\sin\theta =n\;\lambda$$

where *d_hkl_* is the interplaner spacing, *θ* is the angle of diffraction, and *λ* is the wavelength. The grain size/crystallite size was evaluated by following the Scherrer expression [35]:

(3) $$\xi =\frac{0.94\lambda }{\beta cos\theta }$$

where *ξ* represents the crystallite size and *β* is FWHM in radians. The following relation was utilized to evaluate the lattice strain (*ε*) and dislocation density (*δ*).

(4) $$\varepsilon =\frac{\beta }{4tan\theta }$$

(5) $$\delta =\frac{1}{\xi^{2}}$$

A systematic reduction in microstrain and dislocation density was observed for the sample, which was annealed at 300 °C and 400 °C, followed by an increase at 500 °C. The observed decrease in microstrain and dislocation density at 300 °C and 400 °C can be attributed primarily to annealing-induced recovery processes, such as rearrangement and partial annihilation of dislocations. At 500 °C, the observed increase in microstrain and dislocation density may result from surface reconstruction or slight re-oxidation of the surface, leading to lattice distortion.

**Figure 3:**
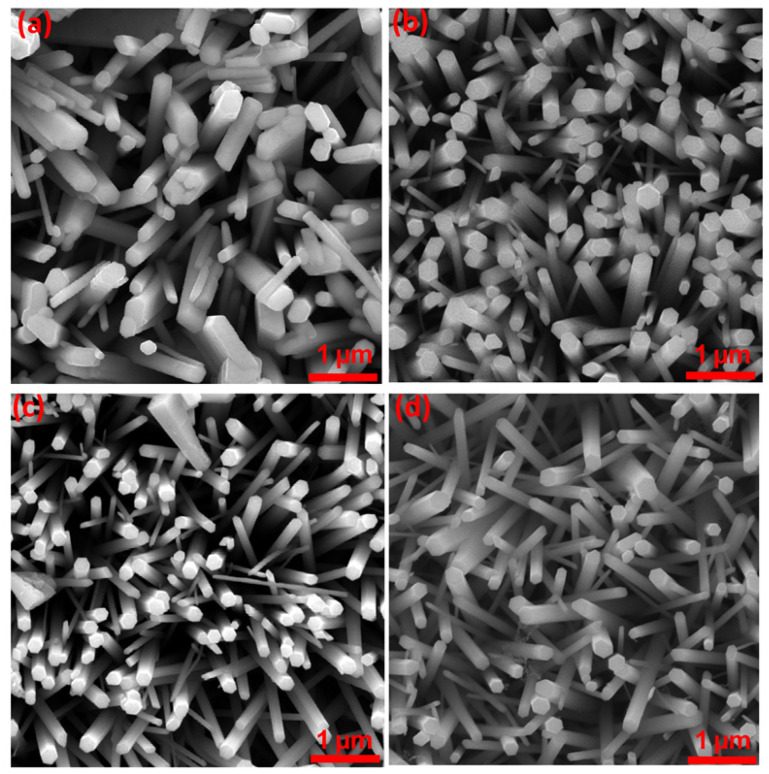
FESEM images of ZnO thin films. (**a**) As-deposited and annealed thin films at (**b**) 300 °C, (**c**) 400 °C, and (**d**) 500 °C.

Figure 3a–d presents the comparative surface characteristics of non-annealed and annealed ZnO thin films. The images reveal that the ZnO thin films consist of nanorods (NRs) oriented vertically on the substrates. A detailed analysis of the FE-SEM images indicates a reduction in nanorod size with increasing annealing temperature. Specifically, the diameter of the nanorods, which is observed without annealing, reduces progressively as the annealing temperature rises, reaching its smallest size at 500 °C. The noticeable decrease in the dimensions of nanorods within the annealed ZnO thin films is primarily influenced by the enhanced rate of nucleation during the thermal treatment [36]. This intensified nucleation mechanism facilitates the early formation of a large number of fine nuclei or primary clusters, as evidenced in Figure 3b–d. The emergence of these abundant small entities effectively reduces the local supersaturation conditions within the system. A lower-supersaturation environment inherently suppresses the thermodynamic driving force for particle enlargement through coalescence, thereby limiting the extent to which individual particles can merge into larger assemblies. This sequence of events illustrates the typical progression involved in the morphological development of ZnO thin films, beginning with the spontaneous initiation of nuclei, followed by the growth of discrete particles, and culminating in the potential, but in this case, inhibited coalescence into larger grain structures. Although a significant variation in the size of nanorods was observed with increasing annealing temperatures, the hexagonal morphology was largely preserved.

**Figure 4:**
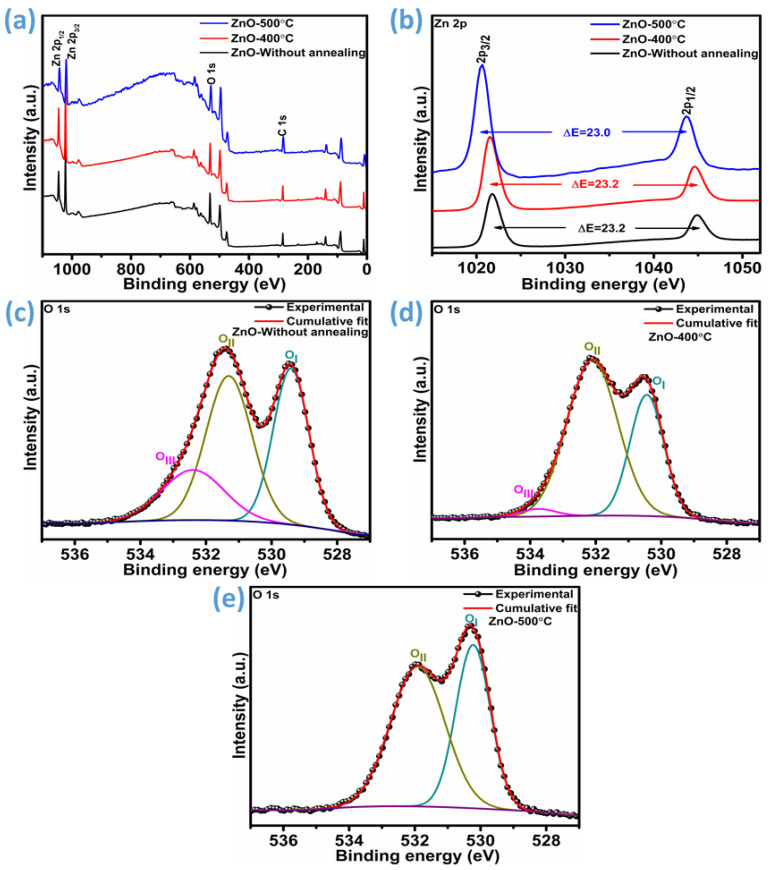
XPS analysis of ZnO thin films. (**a**) Survey spectra of as-deposited and annealed thin film (ZnO-400 °C and ZnO-500 °C), (**b**) Zn 2p spectra of as-deposited and annealed thin film (ZnO-400 °C and ZnO-500 °C), (**c**) O 1s spectra of as-deposited thin film, (**d**) O 1s spectra of annealed thin film (ZnO-400 °C), and (**e**) O 1s spectra of annealed thin film (ZnO-500 °C).

X-ray photoelectron spectroscopy (XPS) was conducted on three samples: unannealed ZnO and ZnO annealed at 400 °C and 500 °C. As illustrated in Figure 4a, the survey spectra reveal well-defined photoelectron and Auger peaks associated with zinc and oxygen. These results confirm that Zn and O are the principal elements present in all samples, with no evidence of detectable impurities, affirming the chemical purity of the synthesized nanoparticles. Figure 4b presents the Zn 2p peak profiles for both the unannealed and annealed ZnO samples, showing two clearly resolved core levels: 2p_3/2_ and 2p_1/2_. The spectral profiles are largely similar; however, in the annealed sample, both core levels exhibit a slight shift toward lower binding energies. The Zn 2p core-level spectra exhibit distinct peaks corresponding to the 2p_3/2_ and 2p_1/2_ states. For the unannealed sample, these peaks are located at 1021.75 eV and 1044.9 eV, respectively. After annealing at 400 °C, the peaks shift slightly to 1021.5 eV (2p_3/2_) and 1044.65 eV (2p_1/2_), indicating a reduction in binding energy of approximately 0.25 eV. Further annealing at 500 °C results in additional shifts, with the 2p_3/2_ peak centered at 1020.6 eV and the 2p_1/2_ peak at 1043.7 eV. These progressive shifts toward lower binding energies suggest subtle modifications in the local chemical environment or electronic structure due to thermal treatment. Despite the observed shifts in absolute binding energies, the spin–orbit energy separation between the 2p_3/2_ and 2p_1/2_ levels remains nearly constant, approximately 23.2 eV for the unannealed and 400 °C samples, and slightly reduced to 23.0 eV after annealing at 500 °C. This characteristic splitting confirms that zinc retains its +2 oxidation state in both samples [37].

To understand the influence of thermal treatment on the O 1s spectral characteristics, peak deconvolution was performed for each ZnO sample. The evolution of the O 1s components with annealing is depicted in Figure 4c–e, corresponding to the pristine ZnO and samples annealed at 400 °C and 500 °C, respectively. For the untreated and 400 °C-annealed samples, the O 1s spectra were resolved into three distinct contributions: lattice oxygen (O–M), oxygen-deficient regions (associated with vacancies), and hydroxyl groups or physically adsorbed water species (O–H) [38]. As observed in Figure 4c,d, the ZnO sample annealed at 400 °C exhibits a notable decrease in the O–H signal. Interestingly, this peak component is entirely absent in the 500 °C-annealed sample, as shown in Figure 4e. In the unannealed ZnO, the O–H group constituted approximately 20.25% of the total O 1s peak area. This value dropped significantly to 2.2% following the 400 °C treatment and was completely eliminated after annealing at 500 °C. Regarding the lattice oxygen (O–M) contribution, a minor decline was observed post-annealing at 400 °C, decreasing from 36.45% to 33.55%. However, upon annealing at 500 °C, this component rose to 43.2%. In contrast, the oxygen vacancy-related signal (O-deficient) showed an increasing trend after initial annealing, rising from 43.3% in the pristine sample to 64.25% at 400 °C, before declining slightly to 56.8% at 500 °C. The increase in oxygen vacancy concentration with moderate annealing is known to positively impact gas sensing performance. This enhancement is attributed to the creation of additional active sites for gas adsorption, an elevated carrier concentration, and improved electron mobility, all of which contribute to superior sensor response characteristics, including heightened sensitivity, faster detection times, and greater selectivity [39,40].

**Figure 5:**
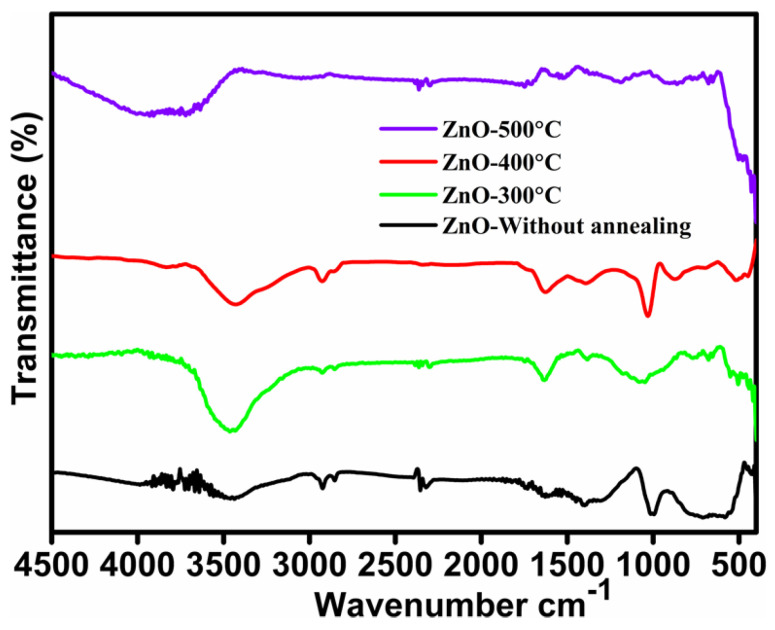
FT-IR spectra of ZnO thin film at various annealing temperatures.

Fourier transform infrared (FTIR) spectroscopy provides crucial insights into the chemical bonding structure and facilitates the identification of elemental constituents within nanomaterials. Figure 5 presents the FTIR spectra of ZnO without annealing and subjected to annealing at various temperatures. The stretching vibrations of hydroxyl (–OH) groups are responsible for the broad absorption band seen in the 3426 cm^−1^-to-3445 cm^−1^ region, which indicates the sample’s moisture content [41]. Furthermore, the presence of organic residues is indicated by a clear peak at 2928 cm^−1^ that is connected to the C–H bond’s stretching vibrations. Additionally, the stretching oscillations of the O=C=O bond are responsible for the band at 2329 cm^−1^, confirming the existence of CO_2_ in the sample. The bending vibrations of water molecules are evident in the absorption peak at approximately 1626 cm^−1^ [42]. Furthermore, the stretching vibrations of carbonyl (C=O) functional groups, which may be affixed to ZnO molecules as a result of surface contacts, are responsible for the spectrum area between 1531 cm^−1^ and 1392 cm^−1^. The production of ZnO nanostructures is further confirmed by the significant peak at 1030 cm^−1^, which is suggestive of asymmetric stretching vibrations of Zn–O bonds [43]. Moreover, the distinctive Zn–O stretching vibrations linked to the wurtzite crystalline structure, a property that distinguishes ZnO, correspond to absorption bands in the 400–600 cm^−1^ wavenumber range.

**Figure 6:**
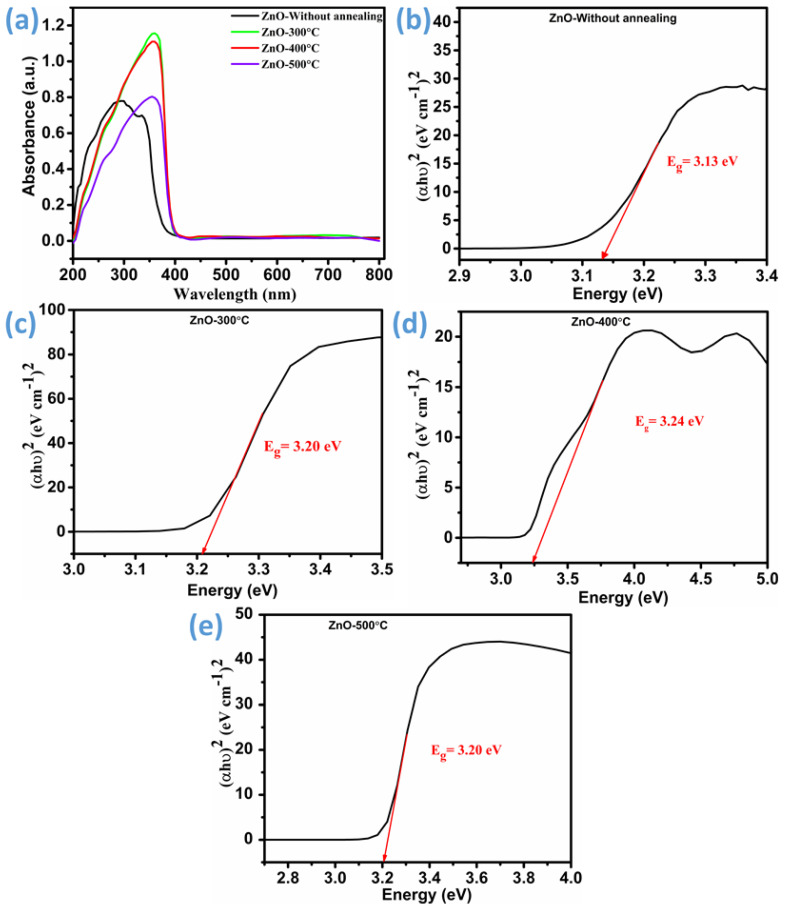
UV-Vis spectrum of ZnO thin films and band gap energy diagrams for as-deposited and annealed ZnO thin-film samples at different temperatures: (**a**) UV-Vis spectrum, (**b**) band gap energy of ZnO without annealing, (**c**) ZnO-300 °C, (**d**) ZnO-400 °C, and (**e**) ZnO-500 °C.

The UV–visible absorption spectra of ZnO thin films, recorded over the 200–800 nm range, are presented in Figure 6a. A prominent absorption edge around 380 nm is observed in all samples, characteristic of the wide-band-gap nature of ZnO. A distinct absorption peak at ~355 nm confirms the presence of ZnO, consistent with its intrinsic excitonic transition. In the as-deposited ZnO thin film, an additional absorption peak appears at approximately 295 nm, which redshifts significantly to 360 nm upon annealing at 500 °C. Notably, the samples annealed at 300 °C and 400 °C exhibit an intermediate absorption peak at ~355 nm with a marked increase in absorption intensity. The redshift of the absorption edge with increasing annealing temperature suggests narrowing of the optical band gap, often associated with band-tailing effects due to a rise in defect concentration. Such defects, particularly oxygen vacancies, play a dual role in gas sensing: they act as active sites for the adsorption of electron-withdrawing gases like NO_2_ and enhance carrier density, facilitating charge transfer at the semiconductor–gas interface [44,45,46]. Therefore, the observed spectral shifts and intensity variations reflect changes in the electronic structure of ZnO induced by annealing, which directly influence its gas sensing properties. At moderate annealing temperatures (300–400 °C), a favorable balance is achieved between defect concentration and crystallinity, promoting enhanced gas adsorption and electron mobility. In contrast, excessive annealing (e.g., at 500 °C) may lead to defect saturation, potentially reducing sensing efficiency.

The band gap energy is provided by the Tauc relation as follows [46]:

(6) $$\alpha h\upsilon =A{\left(h\upsilon -E_{g}\right)}^{n}$$

In this context, ‘α’ denotes the absorption coefficient, ‘h’ represents Planck’s constant, ‘ν’ corresponds to the photon frequency, ‘A’ is a proportionality constant, and E_g_ signifies the band gap energy. The exponent n takes a value of 1/2 for direct transitions and 2 for indirect transitions. Figure 6b–e illustrates the band gap energy of all ZnO thin films. The band gap energy (E_g_) of the ZnO thin film without annealing (Figure 6b) was 3.13 eV, which is less than the values that have been previously reported. This inconsistency is attributed to structural defects introduced during the deposition process, leading to the formation of allowed states near the conduction band within the energy band gap [47]. Upon annealing at 300 °C and 400 °C, a slight increase in the band gap energy was observed. However, beyond 400 °C, a decline in the band gap energy was noted. This reduction is likely caused by thermal ionization defects and an increase in phonon interactions [48]. Excessive thermal energy at higher temperatures may promote defect formation, disrupting the band structure and reducing the optical band gap. This behavior highlights the delicate balance between defect reduction and thermal degradation in annealed ZnO thin films.

**Figure 7:**
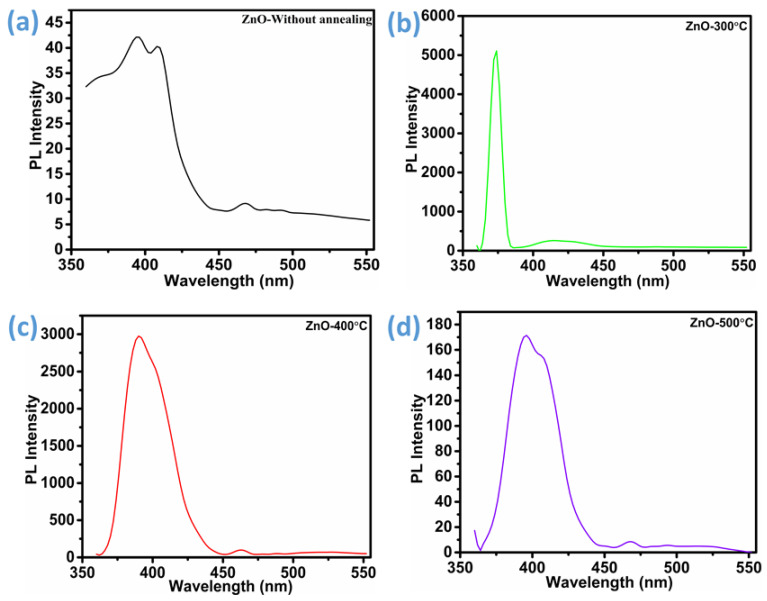
PL spectra of ZnO thin films: (**a**) ZnO-without annealing, (**b**) ZnO-300 °C, (**c**) ZnO-400 °C, and (**d**) ZnO-500 °C.

Photoluminescence (PL) spectroscopy was employed to investigate the optical and defect-related properties of the ZnO thin films, as illustrated in Figure 7a–d. Photoluminescence (PL) analysis revealed that all ZnO samples except the one treated at 300 °C exhibited multiple distinct emission bands. Specifically, the samples annealed at 400 °C and 500 °C displayed three well-resolved emission peaks corresponding to violet, blue, and green luminescence regions. The violet emission was observed in the spectral range of approximately 390–455 nm, the blue emission appeared between 455 and 492 nm, and the green emission was present beyond 500 nm [30]. The violet luminescence exhibited peak positions at 395 nm and 408 nm in both the unannealed ZnO and the sample treated at 500 °C. In contrast, a noticeable blue shift occurred in the ZnO sample annealed at 400 °C, where the corresponding emission peaks appeared at 391 nm and 403 nm. Regarding the blue region, emissions were observed at 468 nm for both the unannealed and 500 °C-treated ZnO, whereas a slight shift to 462 nm was detected in the 400 °C-annealed specimen. As for green emission, it was only faintly detected in the unannealed ZnO, while it became distinctly pronounced around 525 nm in the samples annealed at both 400 °C and 500 °C. These multiple emissions suggest the existence of a complex defect structure within the ZnO lattice, likely influenced by the annealing temperature. In contrast, the ZnO sample annealed at 300 °C showed markedly different behavior, with only a single emission peak detected in the violet region, which also had a blue shift and was centered at 375 nm, while another peak was centered at 412 nm. This limited emission response indicates a relatively lower density or diversity of luminescent defect states in the lower-temperature-treated sample. In general, violet luminescence is attributed to radiative defects, the blue luminescence region reflects single ionized Zn vacancies, and green luminescence reflects the oxygen vacancies [30,49,50,51]. Moreover Wang et al. [52] mentioned that blue emission is also responsible for oxygen vacancies, as oxygen vacancies are known to introduce two distinct donor-type defect states within the electronic band structure of the material. One of these is a shallow-donor level, typically situated approximately 0.3 to 0.5 electron volts (eV) below the conduction band minimum. The total energy separation between this shallow-donor state and the valence band maximum is around 2.8 eV. This energy range aligns closely with the photon energy associated with the blue photoluminescence. Hence, it is reasonable to infer that the observed blue emission arises due to an electronic transition from the shallow-donor state induced by oxygen vacancies down to the valence band.

A detailed examination of Figure 3a,c,d reveals a distinct trend in luminescence behavior across the ZnO samples subjected to different thermal treatments. Among the three, the ZnO sample annealed at 400 °C exhibits the most pronounced emission intensities, in both the blue and green spectral regions. In comparison, the sample treated at 500 °C shows relatively lower but still significant luminescence, while the as-prepared (unannealed) ZnO displays the weakest emissions in these regions. A similar pattern is observed for violet emission, where intensity decreases in the same order: ZnO-400 °C > ZnO-500 °C > unannealed ZnO. The elevated luminescence intensity, particularly in the ZnO-400 °C sample, indicates a higher concentration of intrinsic point defects, such as oxygen vacancies or zinc interstitials, which makes it favorable for gas sensing applications [36,51]. This inference is corroborated by the X-ray photoelectron spectroscopy (XPS) analysis, which further validates the increased defect density in the 400 °C-annealed ZnO. Therefore, the PL response reflects an underlying trade-off between structural perfection and functional-defect-mediated sensing performance. Optimal annealing conditions must balance these effects to ensure enhanced gas–solid interactions without excessively sacrificing material crystallinity.

**Figure 8:**
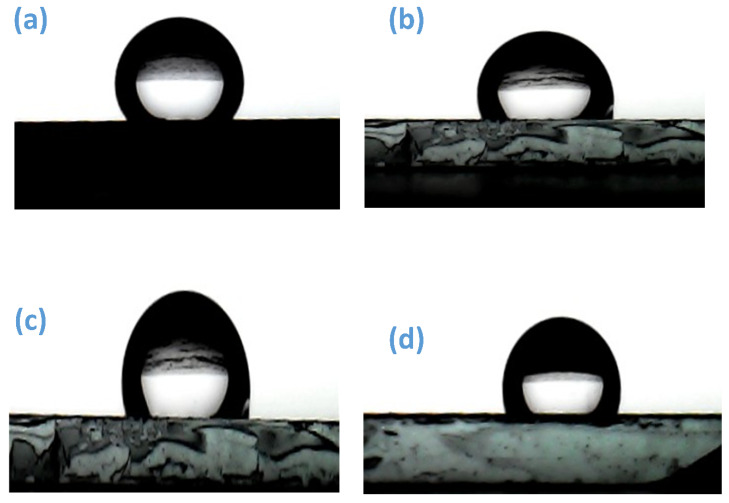
Water droplet contact angle (WCA) images: (**a**) as-deposited and ZnO films annealed at (**b**) 300 °C, (**c**) 400 °C, and (**d**) 500 °C temperatures.

Contact angle measurements are essential for analyzing the surface-wetting behavior of thin films, as illustrated in Figure 8a–d. As a key parameter in surface research, the contact angle provides insights into surface energy. Various factors, including local inhomogeneity and the surface morphology of semiconductor nanomaterials, can influence its value. The interactions of surface free energy at the solid–liquid, solid–gas, and liquid–gas interfaces determine the actual water contact angle. As previously reported [45], the potential of vicinal surfaces for gas sensor applications has been explored. Figure 8a–d present photographic images depicting the shape of water droplets on ZnO thin-film surfaces. The ZnO thin films demonstrate notable hydrophobic behavior, as evidenced by contact angle measurements exceeding 90° for all samples, as presented in Table 2. Interestingly, the contact angle was observed to increase with rising annealing temperature up to 400 °C, indicating enhanced hydrophobicity; however, it decreased again upon further annealing at 500 °C. The conical elongation induced by heat treatment leads to air entrapment within surface gaps, preventing water adhesion to the film. This effect enhances the hydrophobic properties of the ZnO thin-film surface [45]. Surface wettability is fundamentally governed by surface energy, chemical composition, and defect state factors, which collectively play a crucial role in gas adsorption and sensor performance. NO_2_, as a strongly polar and electron-withdrawing gas, exhibits enhanced adsorption on hydrophilic surfaces due to favorable dipole–dipole and hydrogen bonding interactions, thereby improving sensitivity. In contrast, hydrophobic surfaces typically exhibit weaker interactions with polar gases like NO_2_, potentially limiting adsorption efficiency. However, under ambient conditions where humidity is present, hydrophobic surfaces offer a distinct advantage by suppressing water adsorption. This minimizes competition between water and target gas molecules for active sites, thereby preserving sensor functionality. Furthermore, reduced water accumulation on hydrophobic surfaces facilitates more efficient interactions between NO_2_ and the sensing layer, which enhances the chemiresistive response through quicker and more significant changes in electrical resistance [53,54,55].

**Figure 9:**
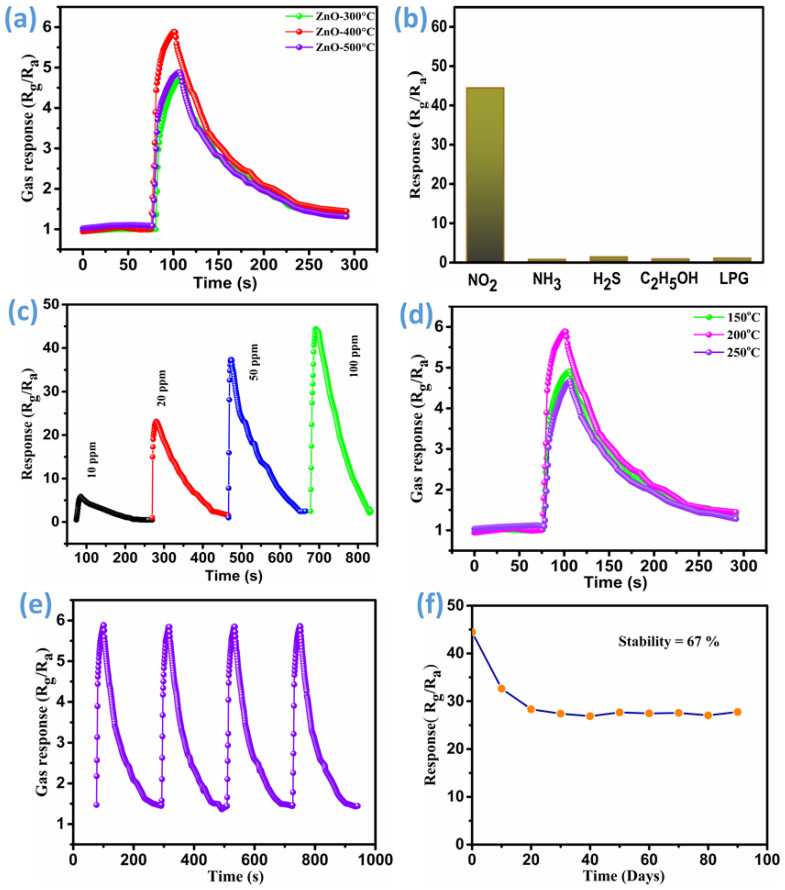
NO_2_ sensing characteristics: (**a**) sensing performance of all ZnO thin films towards 10 ppm of NO_2_ gas at 200 °C, (**b**) analysis of selectivity for a thin film annealed at 400 °C in response to various gases at a concentration of 100 ppm at 200 °C, (**c**) gas response of ZnO-400 sensor for different NO_2_ concentrations at 200 °C, (**d**) sensing properties of ZnO thin film annealed at 400 °C at different operating temperatures, (**e**) reproducibility analysis of ZnO thin film annealed at 400 °C at 10 ppm of NO_2_ gas, and (**f**) stability analysis of ZnO thin film annealed at 400 °C up to 90 days.

When the ZnO sensor interacts with air, it absorbs oxygen molecules onto its surface as negatively charged ions due to the release of free charge carriers from the n-type ZnO. The resistivity of ZnO thin films is increased by this interaction. The annealed ZnO thin-film sensors’ sensitivity measurements, response times, and recovery times are shown in Figure 9a. These two parameters are crucial in gas sensor performance. The recovery time is the amount of time needed for the sensor to return to 10% of its initial reaction, whereas the response time is the amount of time needed for the sensor to reach 90% of its entire response to the gas. These properties depend on a number of variables, including the gas concentration, the operating temperature, and the strength of the link between the gas molecules and the sensor surface. For NO_2_ detection, the response and recovery times of ZnO sensor films annealed at 300 °C, 400 °C, and 500 °C are 17 and 120 s; 13 and 117 s; and 15 and 123 s, respectively. The highest sensitivity of 5.88% at 10 ppm NO_2_ was recorded for the ZnO thin film annealed at 400 °C at an operating temperature of 200 °C, corresponding to a response time of 13 s and a recovery time of 117 s. Compared to previously described NO_2_ gas sensors that use metal oxides as sensing materials, the ZnO thin film annealed at 400 °C demonstrates enhanced gas sensing performance, making it a more effective NO_2_ sensor [56,57,58].

ZnO films annealed at 400 °C demonstrate enhanced sensitivity to NO_2_ due to their optimized crystalline quality and defect structure. Annealing at this temperature reduces structural defects such as dislocations, as noted through the XRD analysis. At this annealing temperature, the ZnO thin film exhibits a mixed preferential orientation, with dominant growth along the (200) plane and partial alignment toward the (100) and (101) planes. The presence of the (100)/(101) planes, known for their higher surface energy and reactivity, enhances the density of active adsorption sites, thereby facilitating stronger interaction with NO_2_ molecules and improving the sensing response. However, further increasing the annealing temperature to 500 °C shifts the crystallographic orientation again largely toward the (100)/(101) planes, which may result in significantly reducing the contribution of the (002) plane. This reorientation is associated with a decline in sensing performance, likely due to excessive grain coarsening, diminished carrier mobility, and a potential decrease in the surface defects. These effects collectively reduce the number and effectiveness of adsorption sites, impairing gas–solid interactions [31]. Additionally, XPS analysis of the ZnO sample annealed at 400 °C indicates an increased concentration of oxygen vacancies, which serve as electron-rich active sites, enhancing NO_2_ adsorption and electron transfer. Complementary surface wettability measurements show that this sample also exhibits the highest degree of hydrophobicity among the tested ZnO films. This hydrophobic characteristic minimizes water accumulation under ambient (humid) conditions, thus reducing interference from moisture and preserving active surface sites for NO_2_ interaction. Together, the optimized crystallographic orientation, increased oxygen deficiency, and favorable surface wettability contribute synergistically to the superior gas sensing performance of ZnO annealed at 400 °C.

The response of the ZnO thin-film sensor annealed at 400 °C is shown in Figure 9b, exhibiting minimal fluctuation when exposed to NH_3_, H_2_S, C_2_H_5_OH, and LPG gases at a concentration of 100 ppm. This indicates a negligible reaction, likely due to the lack of favorable interactions between these gases and the ZnO thin-film surface. Compared to NO_2_ gas, the sensor’s response to other gases is significantly weaker. Therefore, the ZnO thin film with a nanorod array-like morphology demonstrates superior selectivity for NO_2_ gas over other tested gases. The response curve of the ZnO thin-film sensor, which was made using a thin film that was annealed at 400 °C, is shown in Figure 9c as a function of different NO_2_ concentrations at 200 °C. At 200 °C and 10, 20, 50, and 100 parts per million of NO_2_ gas, the sensor’s response values are 5.88%, 23.17%, 37.35%, and 44.45%, respectively. These findings demonstrate that when NO_2_ gas concentrations rise, so does the ZnO thin-film sensor’s sensitivity.

As a function of operation temperature, the ZnO thin-film sensor’s (ZnO@400 °C) sensing response for different gases was assessed. Figure 9d summarizes the results obtained at different temperatures while maintaining constant analyte concentrations. This study examined how the ZnO thin film’s sensing capabilities changed throughout a temperature range of 150 to 250 °C while being continuously exposed to 10 ppm NO_2_. At the ideal operating temperature (200 °C), the sensor responds to its fullest potential. The sensor’s response values were recorded to be 4.90% at 150 °C, 5.88% at 200 °C, and 4.67% at 250 °C. Every subsequent gas sensing test was carried out at 200 °C since this temperature is where the ZnO thin-film sensor performs best of all. This study highlights the higher thermal excitation energies of the ZnO thin-film sensor at 200 °C, which enhance its interaction with adsorbed oxygen species. This interaction facilitates the maximum NO_2_ gas response compared to other operating temperatures. The reproducibility parameter evaluates the reliability of the sensor, while repeatability examines its response to multiple gas exposures, ideally four or more times. Figure 9e illustrates the response of the ZnO thin-film sensor annealed at 400 °C to intermittent injections of 10 ppm NO_2_ gas at an operating temperature of 200 °C. Initially, the sensor exhibits a peak response of 5.88% at 10 ppm and consistently maintains its response upon repeated NO_2_ exposures under identical conditions, confirming its strong repeatability. The stability of the ZnO thin-film sensor annealed at 400 °C was evaluated over a 90-day period at a constant NO_2_ gas concentration of 100 ppm and an operating temperature of 200 °C. As shown in Figure 9f, the sensor’s response gradually decreases from 45% to 30% over this duration, maintaining 67% stability under continuous gas exposure. During the first 15–20 days, the sensor’s response declines rapidly; however, after 20 days, it stabilizes and remains constant over an extended period. This initial decline is likely due to the passivation of the nanomaterial caused by aging.

## 4. Conclusions

This work examines how the annealing temperature affects the optical, morphological, and structural characteristics of ZnO thin films made using the chemical bath deposition method. XRD analysis confirms that the deposited ZnO thin films, comprising nanorods, exhibit a structural orientation depending on the annealing temperature. Quenching of diameter was indicated by the FE-SEM analysis, while UV-Vis measurement indicated an increase in band gap with rising annealing temperature. Gas sensing studies reveal that ZnO films annealed at 400 °C exhibit excellent sensitivity and high selectivity toward NO_2_ gas. This gas sensor also exhibits outstanding stability, repeatability, and improved sensing capabilities at various operating temperatures and NO_2_ gas concentrations. In conclusion, the superior gas sensing performance of ZnO annealed at 400 °C is attributed to its optimized crystal orientation, increased oxygen vacancies, and enhanced surface hydrophobicity, which together promote effective NO_2_ adsorption while minimizing moisture interference under ambient conditions.

## Figures and Tables

**Table 1:** Structural parameters of ZnO thin films.

Sample	2*θ*	FWHM (*β*)	Crystallite Size *ξ* (nm)	Micro Strain (*ε*)	Dislocation Density (*δ*)
As Deposited	34.38	0.23949	34.76	3.231 × 10^−4^	8.257 × 10^−4^
300 °C	34.40	0.2213	37.60	2.989 × 10^−4^	7.070 × 10^−4^
400 °C	34.40	0.2226	37.37	3.008 × 10^−4^	7.158 × 10^−4^
500 °C	34.39	0.2520	33.02	3.403 × 10^−4^	9.169 × 10^−4^

**Table 2:** Contact angle measurement.

Sr. No.	ZnO Thin Film	Contact Angle
1	As deposited	99°
2	Annealed at 300 °C	112°
3	Annealed at 400 °C	116°
4	Annealed at 500 °C	91°

## Data Availability

The original contributions presented in this study are included in the article. Further inquiries can be directed to the corresponding author.
